# Neurodegeneration Upon Dysfunction of Endosomal/Lysosomal CLC Chloride Transporters

**DOI:** 10.3389/fcell.2021.639231

**Published:** 2021-02-23

**Authors:** Shroddha Bose, Hailan He, Tobias Stauber

**Affiliations:** ^1^Institute for Chemistry and Biochemistry, Freie Universität Berlin, Berlin, Germany; ^2^Department of Pediatrics, Xiangya Hospital, Central South University, Changsha, China; ^3^Department of Human Medicine and Institute for Molecular Medicine, MSH Medical School Hamburg, Hamburg, Germany

**Keywords:** autophagy, chloride transport, endosome, ion homeostasis, lysosome, neurodegeneration

## Abstract

The regulation of luminal ion concentrations is critical for the function of, and transport between intracellular organelles. The importance of the acidic pH in the compartments of the endosomal-lysosomal pathway has been well-known for decades. Besides the V-ATPase, which pumps protons into their lumen, a variety of ion transporters and channels is involved in the regulation of the organelles' complex ion homeostasis. Amongst these are the intracellular members of the CLC family, ClC-3 through ClC-7. They localize to distinct but overlapping compartments of the endosomal-lysosomal pathway, partially with tissue-specific expression. Functioning as 2Cl^−^/H^+^ exchangers, they can support the vesicular acidification and accumulate luminal Cl^−^. Mutations in the encoding genes in patients and mouse models underlie severe phenotypes including kidney stones with *CLCN5* and osteopetrosis or hypopigmentation with *CLCN7*. Dysfunction of those intracellular CLCs that are expressed in neurons lead to neuronal defects. Loss of endosomal ClC-3, which heteromerizes with ClC-4, results in neurodegeneration. Mutations in ClC-4 are associated with epileptic encephalopathy and intellectual disability. Mice lacking the late endosomal ClC-6 develop a lysosomal storage disease with reduced pain sensitivity. Human gene variants have been associated with epilepsy, and a gain-of-function mutation causes early-onset neurodegeneration. Dysfunction of the lysosomal ClC-7 leads to a lysosomal storage disease and neurodegeneration in mice and humans. Reduced luminal chloride, as well as altered calcium regulation, has been associated with lysosomal storage diseases in general. This review discusses the properties of endosomal and lysosomal Cl^−^/H^+^ exchange by CLCs and how various alterations of ion transport by CLCs impact organellar ion homeostasis and function in neurodegenerative disorders.

## Introduction

A plethora of ion channels and transporters are responsible for the establishment and maintenance of particular ion concentrations within cells and their membrane-bounded organelles. This ion homeostasis is essential for cellular physiology and its perturbation may lead to dysfunction and eventually to cell death. As long-living post-mitotic cells, neurons are particularly reliant on a functioning endosomal-lysosomal system because of its function in cellular clearance and stress sensing (Boland et al., 2018; Mallucci et al., 2020; Nixon, 2020). Numerous pumps, transporters and channels also mediate the transport of ions required for the physiological functions of these compartments in the endosomal-lysosomal pathway and their dysfunction often underlies inherited disorders of various kinds (Xu and Ren, 2015; Xu et al., 2015; Astaburuaga et al., 2019; Huizing and Gahl, 2020). A key player in the ion homeostasis of endosomes and lysosomes, as well as synaptic vesicles, is the V-ATPase. This multi-subunit enzyme pumps protons into the vesicular lumen and generates the acidic internal pH that regulates enzyme activities, secondary active transport, and membrane trafficking (Mellman et al., 1986; Marshansky and Futai, 2008; Mindell, 2012). Besides the pH, calcium ions (Ca^2+^) have been shown to be of pivotal importance to fusion and fission processes and signaling from endosomes and lysosomes (Luzio et al., 2007; Morgan et al., 2011; Lakpa et al., 2020). For the other major inorganic ions, sodium (Na^+^), potassium (K^+^) and chloride (Cl^−^) it has become clearer during the last years that they may play roles beyond merely provide for electrical and osmotic balance of the organelles. Data from patients, mouse models and cell biophysical measurements highlight an important role for Cl^−^ which is accumulated in a secondary active transport by CLC Cl^−^/H^+^ exchangers (Jentsch, 2007; Stauber and Jentsch, 2013; Schwappach, 2020). Loss, dysfunction or mutations altering biophysical properties of these CLCs lead to severe phenotypes, including neurological defects and neurodegeneration for those CLCs expressed in neurons (Stauber et al., 2012; Jentsch and Pusch, 2018).

In this review we will discuss the physiological roles of the CLC Cl^−^/H^+^ exchangers in the endosomal-lysosomal pathway and their involvement in neuropathologies including neurodegeneration.

## The CLC Family of Cl^−^ Channels and Cl^−^/H^+^ Exchangers

The most abundant physiological anion is chloride. Like for other ions, there are various chloride channels and transporters that conduct chloride, either alone or in symport with, or exchange for other ions. Among these are the members of the CLC family, which notably comprises both chloride channels and chloride/proton exchangers (Stauber et al., 2012; Jentsch and Pusch, 2018). In mammals, there are nine CLC family members that can be divided in three groups by their sequence homology. The first branch contains ClC-1, ClC-2, ClC-Ka, and ClC-Kb, all chloride channels localized to the plasma membrane. By contrast, ClC-3, ClC-4, and ClC-5 of the second group and lastly ClC-6 and ClC-7 function as chloride/proton exchangers on intracellular compartments, mainly in the endosomal/lysosomal pathway.

**Figure 1:**
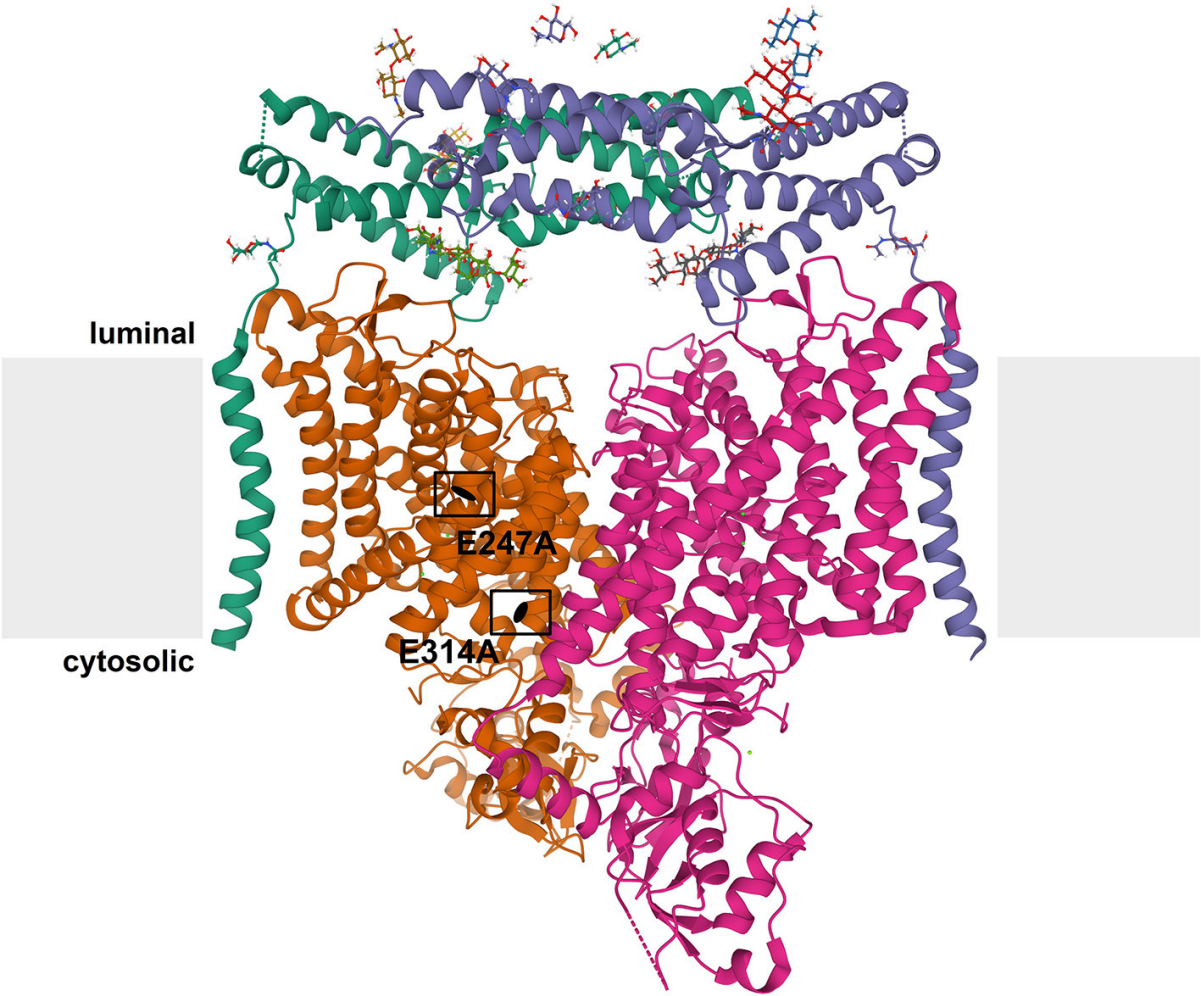
Structure of CLC exchangers. The structure of human ClC-7 in complex with Ostm1 [Protein Data Bank [PDB]: 7JM7 (Schrecker et al., 2020)] viewed parallel to the membrane (depicted in gray) is shown as an example for the structure of CLC proteins. The subunits of the CLC homodimer are represented in orange and magenta. The globular CBS-containing domain of each subunit protrudes into the cytoplasm. Each CLC subunit provides an independent ion transport pathway. For one subunit, the positions of two key amino acids, the “gating glutamate” (E247 in ClC-7) and the “proton glutamate” (E314 in ClC-7), are indicated. The ClC-7 dimer binds two copies of its β-subunit, Ostm1, presented in green and blue. The heavily glycosylated Ostm1 is thought to shield ClC-7 from acidic proteases in the lysosomal lumen.

CLCs function as dimers–mostly homomeric, but some CLCs can heteromerize within the same homology branch–with two independent translocation pathways (Jentsch and Pusch, 2018). Each subunit is composed of a transmembrane domain with a complex structure of multiple alpha helices spanning or penetrating the membrane (Dutzler et al., 2002), and a globular cytosolic domain containing two cystathionine-β-synthase (CBS) domains (Figure 1). The CBS domains of some CLCs can bind adenine nucleotides and may be involved in the regulation and common gating of the transporter or channel (Meyer et al., 2007; Ludwig et al., 2013; Jentsch and Pusch, 2018; Grieschat et al., 2020; Schrecker et al., 2020). Some CLCs additionally bind accessory proteins. Barttin serves as a β-subunit for ClC-Ka and –kb (Estévez et al., 2001) and more recently, an interaction with ClC-5 has been reported (Wojciechowski et al., 2018). ClC-2 and ClC-7 bind to GlialCAM and Ostm1, respectively, that contribute to the subcellular localization, protein stability or ion transport activity of the CLCs (Lange et al., 2006; Leisle et al., 2011; Jeworutzki et al., 2012). A conserved glutamate residue in the ion translocation pathway is critically involved in the gating of the plasma membrane CLC channels and hence referred to as a “gating glutamate” (Jentsch and Pusch, 2018). In the intracellular CLC exchangers, this glutamate is crucial for the strong outward rectification and for the coupling of Cl^−^ transport to H^+^ countertransport. CLC exchangers typically (there are some exceptions in other species) possess a further glutamate, referred to as “proton glutamate,” whose mutation abolishes or strongly diminishes the transport of both protons and chloride (Jentsch and Pusch, 2018; Pusch and Zifarelli, 2021).

Among the CLC channels of the plasma membrane, ClC-1 is expressed in skeletal muscle where it mediates the major resting conductance and hence is involved in the control of muscular excitability (Steinmeyer et al., 1991; Stauber et al., 2012; Jentsch and Pusch, 2018). The homologous ClC-K isoforms are, together with their β-subunit barttin, involved in transepithelial transport in the nephron, inner ear and salivary glands (Jentsch and Pusch, 2018). The broadly expressed ClC-2 plays diverse physiological roles by regulating transepithelial transport, extracellular ion homeostasis and cellular excitability (Stauber et al., 2012; Jentsch and Pusch, 2018). The physiological importance of these plasma membrane chloride channels is evident from patients with mutations of the coding genes and from the phenotypes of engineered mouse models. In case of the ClC-2 knock-out mouse, the phenotype includes a degeneration of the testes and retina leading to male infertility and blindness, and leukodystrophy (Bösl et al., 2001; Nehrke et al., 2002; Blanz et al., 2007; Cortez et al., 2010). Loss-of-function mutations of ClC-2 have been described for some leukodystrophy patients (Depienne et al., 2013; Guo et al., 2019). The two proteins GlialCAM und MLC1, whose mutations can underlie the leukodystrophy megalencephalic leukoencephalopathy with subcortical cysts (MLC) (Leegwater et al., 2001; Lopez-Hernandez et al., 2011), interact with ClC-2 in glia cells and affect the localization and biophysical properties of the chloride channel (Jeworutzki et al., 2012; Hoegg-Beiler et al., 2014). The molecular mechanism leading to leukodystrophy remains to be elucidated, but changes in ClC-2 gating (Jeworutzki et al., 2014) do not seem to be involved (Göppner et al., 2020).

**Figure 2:**
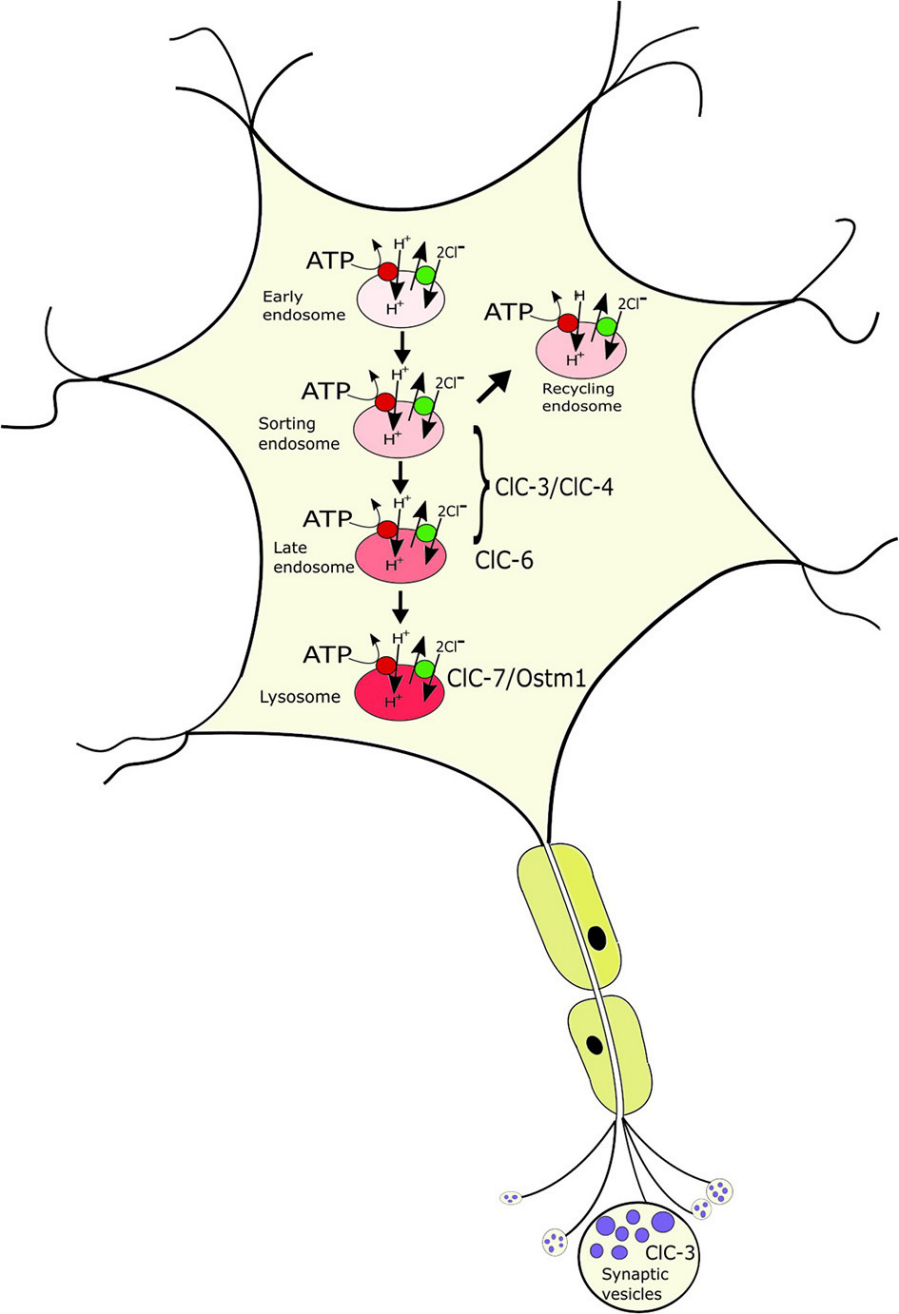
Localization of the neuronal intracellular CLCs to distinct, yet overlapping compartments of the endosomal-lysosomal pathway. ClC-3/ClC-4 localize to various endosomal populations; ClC-6 to late endosomes where it partially overlaps with ClC-7/Ostm1, the only CLC on lysosomes. ClC-3 is to a minor extent additionally found on synaptic vesicles. The CLCs exchange 2Cl^−^ for 1H^+^ and mediate the accumulation of luminal Cl- in the progressively acidified organelles.

ClC-3 through ClC-7 reside on intracellular organelles, predominantly of the endosomal-lysosomal pathway, with a differential distribution between the compartments (Figure 2) (Suzuki et al., 2006; Wartosch et al., 2009; Stauber et al., 2012; Stauber and Jentsch, 2013; Jentsch and Pusch, 2018): ClC-5, which is mainly expressed in the kidney, localizes to early endosomes where it is involved in endocytic uptake in the proximal tubule; the ubiquitously expressed ClC-3 and ClC-4 localize to various endosome populations; ClC-6, which at protein level is almost exclusively expressed in neurons, resides on late endosomes; ClC-7 localizes together with its β-subunit Ostm1 to lysosomes in all cells and additionally to the ruffled border of bone-resorbing osteoclasts. The physiological importance of the intracellular CLCs is evident from the broad spectrum of disorders resulting from their dysfunction in patients and mouse models (Stauber et al., 2012; Jentsch and Pusch, 2018; Schwappach, 2020). Neuronal phenotypes, including intellectual disability, epilepsy, lysosomal storage and neurodegeneration with ClC-3/-4/-6 and−7, respectively (Stobrawa et al., 2001; Kasper et al., 2005; Poët et al., 2006; Veeramah et al., 2013; Hu et al., 2016; Palmer et al., 2018; Nicoli et al., 2019; Polovitskaya et al., 2020), will be discussed in more detail below. In addition, mutations in ClC-5 lead to impaired endocytosis in the renal proximal tubule and kidney stones (Lloyd et al., 1996; Piwon et al., 2000). Mutations in ClC-7 and the associated Ostm1 underlie osteopetrosis due to its role in osteoclast bone resorption (Kornak et al., 2001; Chalhoub et al., 2003) and to impaired skin pigmentation (Nicoli et al., 2019).

ClC-3 though ClC-7 were at first thought to be chloride channels like their plasma membrane-localized homologs that mediate the import of negative charge to counterbalance the electrogenic acidification of the respective organelles (Günther et al., 1998; Piwon et al., 2000; Kornak et al., 2001). However, after the discovery that the bacterial EcClC-1 actually mediates 2Cl^−^/H^+^ exchange (Accardi and Miller, 2004), this transport mode has been shown for the five vesicular CLCs as well (Picollo and Pusch, 2005; Scheel et al., 2005; Graves et al., 2008; Matsuda et al., 2008; Neagoe et al., 2010; Leisle et al., 2011; Guzman et al., 2013). It was proposed that instead of merely allowing Cl^−^ influx to allow for acidification by the V-ATPase, ClC-5 and ClC-3 actively acidified early endosomal compartments by exchanging the initially high luminal chloride for protons (Smith and Lippiat, 2010; Rohrbough et al., 2018). This transport direction would be easier to reconcile with the strong outward rectification, which is observed for all vesicular CLCs upon heterologous expression at the plasma membrane (Friedrich et al., 1999; Li et al., 2000; Neagoe et al., 2010; Leisle et al., 2011) because this would favor Cl^−^ efflux from the lumen through the CLC when localized intracellular vesicles. However, it is questionable whether such a mechanism would effectively acidify the lumen with the typical buffering capacity and it would quickly build up an inside positive potential that would prevent further Cl^−^ efflux through the CLC (Stauber et al., 2012). For ClC-3, it has been shown that low extracytosolic pH partially uncouples Cl^−^ transport from H^+^ exchange (Rohrbough et al., 2018). The authors proposed that at the luminal low pH, ClC-3 would then function as a Cl^−^ conductance that provided the countercharge for further acidification by the V-ATPase. However, more recently, the proton-activated chloride channel PAC has been shown to mediate Cl^−^ efflux from acidified endosomes (Osei-Owusu et al., 2021). The increase in endosomal [Cl^−^] and reduced pH upon its depletion (Osei-Owusu et al., 2021) are compatible with the parallel presence of a CLC exchanger that mediates pH gradient-driven Cl^−^ influx (Jentsch, 2007; Stauber and Jentsch, 2013). For lysosomes, Cl^−^ accumulation by the ClC-7 Cl^−^/H^+^ exchanger has indeed been shown (Weinert et al., 2010). The electrogenic 2Cl^−^/H^+^ exchange can of course still support luminal acidification, and this seems to be an important role for ClC-5 on early endosomes (Novarino et al., 2010). By contrast, the acidification of lysosomes does not necessarily depend on parallel Cl^−^ influx as it can be supported by cation efflux (Steinberg et al., 2010; Weinert et al., 2010). In addition to the acidification of early endosomes, a pivotal role of the vesicular CLCs is the pH gradient-dependent secondary active accumulation of luminal Cl^−^, as highlighted in engineered mouse models in which individual CLC exchangers were mutated to pure chloride conductors (Novarino et al., 2010; Weinert et al., 2010, 2020). The importance of lysosomal Cl^−^ is still elusive, but it may affect further parameters of the vesicular ion homeostasis including Ca^2+^ (Stauber and Jentsch, 2013; Chakraborty et al., 2017; Astaburuaga et al., 2019).

## Neuropathies With ClC-3 and ClC-4

### ClC-3/ClC-4 Mediate Endosomal Cl^–^/H^+^ Exchange in Neurons

Within their branch of CLCs, ClC-3, ClC-4 and ClC-5 share sequence identity of approximately 80%. Whilst ClC-5 is mainly expressed in the kidney (Steinmeyer et al., 1995), ClC-3 is widely expressed and has been detected in virtually all mammalian tissues, including brain, retina, adrenal gland, heart, liver, kidney, pancreas, intestines, epididymis and skeletal muscle (Kawasaki et al., 1994; Borsani et al., 1995; Stobrawa et al., 2001; Maritzen et al., 2008). Within the brain, ClC-3 is predominantly expressed in neuronal cells of the hippocampus and Purkinje cells in the cerebellum (Kawasaki et al., 1994). ClC-3 predominantly localizes on endosomes (Stobrawa et al., 2001; Hara-Chikuma et al., 2005; Shibata et al., 2006). Additionally, it is found on synaptic vesicles (SVs) (Stobrawa et al., 2001; Salazar et al., 2004; Weinert et al., 2020) and synaptic-like micro vesicles (Salazar et al., 2004; Maritzen et al., 2008). ClC-4 displays similarly broad tissue expression as ClC-3, but its relative abundance is lower in tissues other than brain and muscle (Van Slegtenhorst et al., 1994; Mohammad-Panah et al., 2002, 2003; Weinert et al., 2020).

Alternative splice variants of ClC-3 have been reported. The isoforms (ClC-3A, ClC-3B, and ClC-3C) display similar transport properties but differential subcellular localization (Guzman et al., 2015). ClC-3A resides on late endosomes/lysosomes (Li et al., 2002; Gentzsch et al., 2003), ClC-3B might localize to the Golgi (Ogura et al., 2002; Gentzsch et al., 2003), and ClC-3C is targeted to recycling endosomes via an amino-terminal sorting motif (Guzman et al., 2015). Upon heterologous expression, ClC-3 through−5 can form heteromers amongst each other, but not with ClC-6 or ClC-7 (Suzuki et al., 2006). In heterologous expression, ClC-4 is predominantly retained in the endoplasmic reticulum (ER), with a minor portion on the plasma membrane (Okkenhaug et al., 2006; Guzman et al., 2017; Weinert et al., 2020). Co-expression with distinct ClC-3 splice variants targets ClC-4 to late endosome/lysosomes (ClC-3A and ClC-3B) or recycling endosomes (ClC-3C) (Guzman et al., 2017). Also endogenous ClC-3 and ClC-4 interact *in vivo*, and ClC-4 requires ClC-3 for ER export and protein stability (Weinert et al., 2020). This, together with the relative expression levels, suggests that in brain, ClC-3/ClC-4 function as heterodimers, whereas in other tissues there may be a more prominent role for ClC-3 homomers. ClC-4, on the other hand, seems to rely on heteromerization with ClC-3, which may explain the apparent lack of endosomal sorting signals in ClC-4 (Stauber and Jentsch, 2010).

There are numerous conflicting studies concerning the transport properties and cellular functions of ClC-3 (Jentsch and Pusch, 2018). For example, previously ClC-3 was reported to function as a plasma membrane chloride channel regulated by the protein kinase C or the calcium/calmodulin-dependent protein kinase II (Kawasaki et al., 1994; Huang et al., 2001). Another function that had been ascribed to ClC-3 was that of the volume-regulated anion channel (VRAC) (Duan et al., 1997). However, like many other candidates, ClC-3 was excluded as VRAC and the typical VRAC currents were unchanged in the various tested cell types from independent ClC-3 knock-out mouse models (Stauber, 2015; Jentsch and Pusch, 2018); VRAC has more recently been shown to be formed by LRRC8 proteins (Qiu et al., 2014; Voss et al., 2014; König and Stauber, 2019). Instead, ClC-3 is an outwardly rectifying, vesicular 2Cl^−^/H^+^ exchanger like the other intracellular CLCs (Stobrawa et al., 2001; Matsuda et al., 2008; Guzman et al., 2013, 2015; Rohrbough et al., 2018). ClC-3 was reported to provide an electrical shunt for the efficient proton pumping of the electrogenic H^+^-ATPase into the lumen, thereby facilitating the acidification of SVs (Stobrawa et al., 2001; Riazanski et al., 2011; Guzman et al., 2013) and compartments in the endosomal/lysosomal pathway (Stobrawa et al., 2001; Hara-Chikuma et al., 2005; Weylandt et al., 2007). Indeed, endosomal acidification and chloride accumulation were significantly enhanced in ClC-3A-transfected Chinese hamster ovary cells and impaired in hepatocytes from ClC-3-deficient mice (Hara-Chikuma et al., 2005). The acidification of SVs derived from ClC-3-deficient *Clcn3*^−/−^ mice was impaired (Stobrawa et al., 2001; Riazanski et al., 2011). However, the impaired acidification of SVs can be attributed to a secondary decrease in the vesicular glutamate transporter VGLUT1, which itself represents a major Cl^−^ permeation pathway in synaptic vesicles (Schenck et al., 2009; Preobraschenski et al., 2014; Eriksen et al., 2016; Martineau et al., 2017). In young *Clcn3*^−/−^ mice, before the onset of neurodegeneration (see below) and the loss of VGLUT1, SV acidification was not impaired (Weinert et al., 2020). The partial plasma membrane localization of ClC-4 allowed its electrophysiological characterization as a strongly voltage-dependent intracellular 2Cl^−^/H^+^ exchanger (Picollo and Pusch, 2005; Scheel et al., 2005). Also for ClC-4, a role in endosomal acidification was reported, with a more alkaline endosomal pH and resultant defects in recycling of the transferrin receptor in fibroblasts derived from ClC-4-deficient mice (Mohammad-Panah et al., 2003, 2009).

### ClC-3/ClC-4 in Neurodegeneration and Other Neurological Disorders

Three independently generated ClC-3 knock-out mouse models displayed similar phenotypes of severe postnatal degeneration of the retina and brain, that led to an almost total loss of the hippocampus after 3 months (Stobrawa et al., 2001; Dickerson et al., 2002; Yoshikawa et al., 2002) (Table 1). Neurodegeneration in these *Clcn3*^−/−^ mice is not associated with obvious deposits of lysosomal storage material found in *Clcn6*^−/−^ or *Clcn*7^−/−^ mice (Kasper et al., 2005; Poët et al., 2006) (see below). However, one *Clcn3*^−/−^ mouse model displayed deposits of the mitochondrial ATP synthase subunit c, typically found in the lysosomal storage disease neuronal ceroid lipofuscinosis (NCL) (Yoshikawa et al., 2002). The neurodegeneration was accompanied by an activation of microglia and astrogliosis (Stobrawa et al., 2001; Dickerson et al., 2002). Despite their severe neurodegeneration, ClC-3 knock-out mice are viable and have a normal life span.

**Table 1:** Mouse models with *Clcn3, Clcn4* or *Clcn5* mutations.

	***Clcn3*^−/−^ (3 independent lines)**	***Clcn3*^unc/unc^**	***Clcn4*^−/−^**	***Clcn3*^unc/unc^; *Clcn4*^−/−^**	***Clcn3*^−/−^*; Clcn4*^−/−^**	***Clcn5^*y*/−^***	***Clcn5^*y*/^*^unc^**
Ion transport by respective CLC	No current	No proton transport	No current	No proton transport/no current	No current	No current	No proton transport
Age at death	>1 year	Normal	Normal	5–10 weeks	Shortly after birth	Normal	Normal
Weight	↓	Normal	Normal	↓		Normal	Normal
Neuro-degeneration	Yes	No	No	Yes (severe)	Yes (very severe)		
Retina degeneration	Yes	No	No	Yes			
ClC-3 protein levels	No	Normal	Normal	Normal	No	Normal	Normal
ClC-4 protein levels	↓	Normal	No	No	No	Normal	Normal
PH of synaptic vesicles	Normal in young mice; ↑ in old mice	Normal	Normal	↑			
Endosomal pH	↑ in hepatocytes; normal in neurons					↑	Normal
Endosomal chloride	↓ in hepatocytes; not tested in neurons						
Others						Impaired renal endocytosis	Impaired renal endocytosis

*See main text for references*.

It is tempting to speculate that the severe neurodegeneration in *Clcn3*^−/−^ mice is caused by impaired synaptic function. The frequency and amplitude of miniature excitatory postsynaptic currents (mEPSCs) in primary neurons from ClC-3-deficient mice was reported to be reduced, suggesting that glutamate toxicity due to excessive glutamate release contributes to the neurodegeneration (Guzman et al., 2014). However, a recent study found virtually unaltered mEPSCs (Weinert et al., 2020). Another study reported reduced miniature inhibitory postsynaptic currents (mIPSCs) in *Clcn3*^−/−^ mice (Riazanski et al., 2011), in agreement with the reduced glutamate uptake in SVs of ClC-3-deficient mice, but in contrast to mIPSC measurements in a previous study (Stobrawa et al., 2001). Both the impaired acidification and the glutamate loading of SVs can be explained by the concomitant reduction of VGLUT1 in *Clcn3*^−/−^ mice (Stobrawa et al., 2001; Schenck et al., 2009; Weinert et al., 2020), which is a likely consequence–and hence not cause–of the neurodegeneration.

A recent study described knock-in mice in which the “gating glutamate” in ClC-3 is replaced by alanine to form the “ClC-3^unc^” mutant, in which Cl^−^ transport is *unc*oupled from H^+^ countertransport (Weinert et al., 2020). These mice, referred to as *Clcn3*^unc/unc^, presented no obvious phenotype (Table 1). This is in stark contrast to previous observations from equivalent mouse models expressing ClC-5^unc^ and ClC-7^unc^ that display similar phenotypes as the respective knock-out mouse models (Novarino et al., 2010; Weinert et al., 2010). The explanation lies in the heteromerization of ClC-3 with ClC-4 (Weinert et al., 2020). Although mRNA levels of ClC-4 were not significantly altered in the brain of *Clcn3*^−/−^ mice, ClC-4 protein levels were decreased to ~30%. In contrast, no reduction in ClC-4 levels was observed in *Clcn3*^unc/unc^ mice. ClC-3^unc^ stabilizes and promotes the transport of ClC-4 from the ER to endosomal compartments like wild-type ClC-3. Therefore, ClC-4 may compensate for a loss of ClC-3 function in *Clcn3*^unc/unc^, but not in Clcn3^−/−^ mice, and the partial loss of ClC-4 may contribute to the severe neurodegeneration of *Clcn3*^−/−^ mice (Weinert et al., 2020). Mice deficient in ClC-4, as well as *Clcn3*^unc/unc^ mice displayed no obvious phenotypes (Rickheit et al., 2010; Hu et al., 2016; Weinert et al., 2020). However, the combination of these genotypes in *Clcn3*^unc/unc^;*Clcn4*^−/−^ mice results in an even more severe neurodegeneration than observed in *Clcn3*^−/−^ mice (Weinert et al., 2020) (Table 1). Yet, the milder phenotype of *Clcn3*^unc/unc^;*Clcn4*^−/−^ mice compared to *Clcn3*^−/−^;*Clcn4*^−/−^ mice suggests that the pure Cl^−^ conductance of the uncoupled ClC-3^unc^ can also partially substitute for functions of the wild-type ClC-3 Cl^−^/H^+^ exchanger in the absence of compensating ClC-4 (Weinert et al., 2020). The absence of neurodegeneration in *Clcn4*^−/−^ mice can be explained by the remaining, unaltered levels of ClC-3 which may be sufficient, as ClC-3 can form homodimers and does not require ClC-4 for its endosomal localization. For the properties of ClC-3, only subtle consequences of the heteromerization with ClC-4 are expected.

Like in *Clcn3*^−/−^ mice, the acidification of SVs and hippocampal miniature postsynaptic currents were unaltered in *Clcn3*^unc/unc^ mice before major neuronal loss, suggesting that the severe neurodegeneration does probably not result from SV dysfunction (Weinert et al., 2020). Only a minor fraction of ClC-3 is found on SVs, while also in neurons the majority is present on endosomes. Endosomal acidification and concomitant Cl^−^ accumulation was found significantly impaired in hepatocytes from ClC-3-deficient mice (Hara-Chikuma et al., 2005). However, pH measurements of transferrin-positive endosomes in cultured neurons from *Clcn3*^−/−^ mice revealed normal acidification (Weinert et al., 2020), resembling findings with lysosomes from mice lacking ClC-7 or Ostm1 (Kasper et al., 2005; Lange et al., 2006) (see below). Mouse models expressing ClC-5^unc^ or ClC-7^unc^ suggested an important role for pH gradient-driven vesicular Cl^−^ accumulation in early endosomes and lysosomes, respectively (Novarino et al., 2010; Weinert et al., 2010). So the severe neurodegeneration observed in *Clcn3*^−/−^ mice or *Clcn3*^unc/unc^*/Clcn4*^−/−^ mice may be ascribed to an impairment of endosomal chloride accumulation rather than to defective endosomal acidification (Weinert et al., 2020).

So far, no convincing disease-causing mutations have been identified in the human *CLCN*3 gene. However, a variety of inherited and *de novo* mutations in *CLCN4*, which in humans is located X-chromosomal in contrast to the autosomal positioning of the *Clcn4* gene in the laboratory mouse *Mus musculus* (Palmer et al., 1995; Rugarli et al., 1995), have recently been identified in patients with intellectual disability, epilepsy and behavior disorders, but lacking neurodegeneration (Veeramah et al., 2013; Hu et al., 2016; Palmer et al., 2018; Zhou et al., 2018). The identified *CLCN4* mutations included frameshifts, missense, intragenic copy number deletion and splice site alterations. *In vitro* electrophysiological studies showed that the majority of the disease-associated missense mutations cause a loss of function as they diminished or abolished the outwardly rectifying ClC-4 currents upon heterologous expression. Although no neurological phenotype and no morphological changes in the brain were detected in *Clcn4*^−/−^ mice (Rickheit et al., 2010; Palmer et al., 2018), the number of dendritic branches and dendritic length were reduced in primary neurons derived from *Clcn4*^−/−^ mice and in knock-down of the *Clcn4* gene cultures neurons (Hur et al., 2013; Hu et al., 2016). This suggests an involvement of ClC-4 in neuronal differentiation, which may contribute to the neurological disorders in patients with *CLCN4* mutations.

## The Late Endosomal ClC-6

ClC-6 and ClC-7, which share ~45% sequence identity, constitute the third branch of mammalian CLCs (Brandt and Jentsch, 1995). Despite the ubiquitous expression of *CLCN6* mRNA (Brandt and Jentsch, 1995; Kida et al., 2001), the protein is almost exclusively found in in neurons (Poët et al., 2006). Whilst upon heterologous expression in cell culture ClC-6 is mainly targeted to recycling endosomes (Ignoul et al., 2007; Stauber and Jentsch, 2010), endogenous ClC-6 resides predominantly on Lamp1-positive late endosomes (Poët et al., 2006), partially overlapping with endosomal ClC-3 and late endosomal/lysosomal ClC-7 (Stauber et al., 2012; Jentsch and Pusch, 2018). The partial plasma membrane localization of ClC-6 with green-fluorescent protein (GFP) fused to its amino-terminus allowed for its biophysical characterization as a Cl^−^/H^+^ exchanger (Neagoe et al., 2010). ClC-6 shares typical properties with the other intracellular CLCs, such as the outward rectification and the uncoupling of Cl^−^ transport from H^+^ countertransport by mutation of the “gating glutamate.”

ClC-6-deficient *Clcn6*^−/−^ mice are fertile and display a normal life pan without any immediately apparent phenotype (Poët et al., 2006) (Table 2). However, their neurons accumulate lysosomal storage material, also containing the mitochondrial ATP synthase subunit c, specifically in the initial axon segments that become swollen (Poët et al., 2006). Detailed analysis of brains from *Clcn6*^−/−^ mice revealed a late-onset—much milder than for ClC-3 or ClC-7 deficiency—neurodegeneration (Pressey et al., 2010). ClC-6 knock-out mice also exhibited minor cognitive defects and reduced pain sensitivity, which may arise from the accumulation of storage material in neurons of the dorsal root ganglia (Poët et al., 2006). While two out of 75 tested patients with Kuf's disease, which features phenotypes in common with those found in ClC-6 knock-out mice (Berkovic et al., 1988), were heterozygous for *CLCN6* missense variants, no further evidence for ClC-6 dysfunction underlying this disease was found (Poët et al., 2006).

**Table 2:** Mouse models with *Clcn6* or *Clcn7* mutations.

	***Clcn6*^−/−^**	***Clcn7*^−/−^**	***Clcn7*^unc/unc^**	***Clcn7*^td/td^**	***Clcn7*^G213R/G213R^**	***Clcn7*^F316L/F316L^**	***Clcn7*^+/Y713A^**
Transporter properties	No ClC-6 protein	No ClC-7 protein	No proton transport, no rectification, instantaneous currents	Only residual currents	Mis-localization	Reduced current	More currents at cytosolic-positive potential
Age at death	Normal	4-6 weeks	Up to 5 weeks	Up to 6 weeks	Up to 30 days	Up to 30 days	18 weeks (heterozygous)
Fur color	Normal	Gray	Normal	Normal			Albinism
Bone mineralization		↑	↑	↑	↑	↑	Normal
Storage material	Yes	Yes	Yes	Yes	Yes		Yes
Neuro-degeneration	No	Yes	Yes	Yes	Yes		Yes
Retina degeneration		↑	↑	No			
Autophagic accumulation		↑	↑	No	↑		
Lysosomal pH	Normal	Normal	Normal	Normal	↑		↓
Lysosomal chloride		↓	↓	↓			
Others	Reduced pain sensitivity, moderate behavioral abnormalities	Splenomegaly			Fibrosis in lung, kidney, muscle		Intracellular vacuoles

*See main text for references*.

Variations in *CLCN6* were identified in patients with epilepsy (Yamamoto et al., 2015; Wang et al., 2017; Peng et al., 2018; He et al., 2021). Interestingly, these include a mutation of the “gating glutamate” at position E200 in the commonly used *CLCN6* transcript variant 1 (He et al., 2021), while the mutation was referred to as E178A in earlier studies (Wang et al., 2017; Peng et al., 2018). This amino acid substitution converts ClC-6 into a pure Cl^−^ conductor (Neagoe et al., 2010). Interestingly, a recent study shows that this ClC-6^unc^ mutant, identified in a patient with the early infantile epileptic encephalopathy West syndrome, impairs the autophagic-lysosomal pathway upon heterologous expression (He et al., 2021). Very recently, a heterozygous *de nov*o *CLCN6* missense mutation was identified in three independent patients with variable early-onset neurodegeneration with brainstem lesions and cortical or cerebral atrophy, respectively (Polovitskaya et al., 2020). The affected children displayed global developmental delay with regression and further neurological disorders such as peripheral sensory neuropathy to varying degrees, but lacked seizures. The identified mutation, p.Tyr553Cys, affects a tyrosine that is conserved between the mammalian CLC exchangers. The electrophysiological characterization of the ClC-6^Y553C^ mutant revealed that at cytosolic-positive membrane potentials, it mediated larger currents than wild-type ClC-6, which were not affected by an extra-cytosolic pH of 5.5 similar to that in late endosomes. Voltage-dependent activation of the mutant was slowed down in comparison to wild-type, but the instantaneous currents were already of the amplitude of maximal wild-type currents. So this mutation resulted in a gain of function (Polovitskaya et al., 2020). Upon heterologous expression in cultured cells it colocalized with the marker protein Lamp1 to drastically enlarged late endosomal structures (Polovitskaya et al., 2020), resembling those observed with gain-of-function ClC-7 mutant (Nicoli et al., 2019) (see below). The lumen of these vacuoles was poorly acidified (Polovitskaya et al., 2020), which may cause alterations of membrane fusion and fission with or from these structures. The consequent impairment of the endosomal-lysosomal pathway is likely to underlie the neuropathy of the patients.

The two disease-causing mutations have differential effects on the ion transport properties of ClC-6. However, the fact that loss of ClC-6 in *Clcn6*^−/−^ mice leads to only mild phenotypes (Poët et al., 2006), suggests that the heterozygous ClC-6^E200A^, just like ClC-6^Y553C^ (Polovitskaya et al., 2020), presents a gain-of-function mutation. The uncoupling of chloride transport from proton countertransport by the E200A mutation may be considered a loss of function. Indeed, mouse models expressing the equivalent mutation in ClC-5 or ClC-7 display similar disorders as the respective knock-out models (Novarino et al., 2010; Weinert et al., 2010). However, these uncoupling mutations additionally abolish the outward rectification of the CLC and hence would increase currents at luminal positive potentials, which may well be of patho-physiological relevance. Nonetheless, the two ClC-6 mutations also affect the morphology of endosomal compartments differentially –with only mildly or drastically enlarged compartments for ClC-6^E200A^ and ClC-6^Y553C^, respectively (Polovitskaya et al., 2020)– and eventually lead to different neurological disorders, infantile epilepsy or early-onset neurodegeneration.

## Neurodegeneration Upon Dysfunction of ClC-7/Ostm1

### ClC-7/Ostm1—a Lysosomal Cl^–^/H^+^ Exchanger

The ubiquitously expressed ClC-7 is the only member of the CLC protein family that primarily localizes to lysosomes (Brandt and Jentsch, 1995; Kornak et al., 2001). In bone-resorbing osteoclasts, it additionally resides in the ruffled border, a specialized membrane domain built up by the fusion of lysosomes with the plasma membrane (Teitelbaum, 2000; Kornak et al., 2001). It forms a stable complex with the single-pass type I membrane protein Ostm1 (for osteopetrosis-associated membrane protein 1) (Lange et al., 2006; Schrecker et al., 2020; Zhang et al., 2020) (Figure 1). This interaction is required for stability for protein stability of ClC-7, likely due to the heavily N-glycosylated Ostm1, of which two subunits bind to the ClC-7 dimer, shielding the unglycosylated ClC-7 from lysosomal proteases (Lange et al., 2006; Schrecker et al., 2020; Zhang et al., 2020), and for ion transport by the ClC-7/Ostm1 complex (Leisle et al., 2011). On the other hand, Ostm1 is reliant on ClC-7 for exit from the ER and lysosomal targeting (Lange et al., 2006; Leisle et al., 2011). Hence, a drastic reduction in protein levels can be observed for Ostm1 in tissues of ClC-7-deficient *Clcn7*^−/−^ mice and for ClC-7 in spontaneous Ostm1-deficient *grey-lethal* mouse line (Lange et al., 2006). Ostm1 is a 70 kDa protein which is proteolytically processed upon arrival in lysosomes (Lange et al., 2006). Recently solved cryo-EM structures of ClC-7/Ostm1 revealed that the luminal domain of Ostm1 has a tightly packed core of helical bundles linked with several disulfide bonds (Schrecker et al., 2020; Zhang et al., 2020).

In osteoclasts, expression of ClC-7 and Ostm1 are coregulated by the transcription factor microphthalmia (Meadows et al., 2007). In addition, ClC-7 expression has been shown to be upregulated, like that of many lysosomal proteins, by the transcription factor TFEB (Sardiello et al., 2009). The subcellular localization of ClC-7/Ostm1 on late endosomes/lysosomes has been shown by various means for all tested tissues and cell types, including neurons, fibroblasts, renal proximal tubule cells, liver, macrophages, activated microglia and HeLa cells (Kornak et al., 2001; Kasper et al., 2005; Graves et al., 2008; Wartosch et al., 2009; Steinberg et al., 2010; Majumdar et al., 2011; Hennings et al., 2012). Lysosomal localization increases reportedly during microglia activation (Majumdar et al., 2011). Recently, an influence of protein kinase A (PKA) signaling on lysosomal delivery of ClC-7/Ostm1 and an impairment thereof in presenelin-1 knock-out has been proposed (Lee et al., 2020). In neurons, the ClC-7 may partially overlap with ClC-6 in their localization to late endosomes, but ClC-7 is exclusively found on lysosomes to which ClC-6 is only shifted in brain of ClC-7-deficient mice (Poët et al., 2006).

Like for ClC-6, the exclusive intracellular localization of ClC-7 hampered a biophysical characterization of the protein for several years. The presence of a conserved “proton glutamate” (E312 or E314 in mouse and human ClC-7, respectively) pointed to the activity of ClC-7 as a Cl^−^/H^+^ antiporter. This was confirmed by flux measurements on isolated lysosomes (Graves et al., 2008) and with lysosomes in living cells, including ClC-7 knock-out cells as control (Weinert et al., 2010). Subsequently, the identification of endosomal sorting motifs in the amino-terminal domain of ClC-7 enabled partial cell surface localization of ClC-7/Ostm1 (Stauber and Jentsch, 2010). The plasma membrane-targeted ClC-7 mutant depleted of the sorting motifs, referred to as ClC-7^PM^, mediates strongly outward-rectifying voltage-activated currents by exchanging 2 Cl^−^ for 1 H^+^ (Leisle et al., 2011). Both Cl^−^ and H^+^ transport could also be visualized in an optical activity assay for ClC-7/Ostm1 (Zanardi et al., 2013). The electrophysiological analysis revealed that ClC-7/Ostm1 shares many properties with the other intracellular CLCs, including the uncoupling of Cl^−^ transport from proton countertransport and the drastic reduction in transport upon mutation of the “gating” and “proton” glutamates, respectively (Leisle et al., 2011). ClC-7^PM^ required co-expression of Ostm1 for ion transport activity (Leisle et al., 2011), which may be due to minor effects of Ostm1 on the ion translocation pathway in the ClC-7 subunit (Schrecker et al., 2020). A striking difference to the currents mediated by the endosomal CLCs was the slow activation and relaxation kinetics of ClC-7/Ostm1 (Leisle et al., 2011). This slow “gating” involves the common gating of the subunits (Ludwig et al., 2013) and depends on the interaction of the ClC-7 transmembrane domain interface with the amino-terminus and the CBS domains of the C-terminal region (Leisle et al., 2011; Schrecker et al., 2020; Zhang et al., 2020).

### Neurodegeneration in Mouse Models With Dysfunction of ClC-7/Ostm1

The physiological importance of ClC-7/Ostm1 was first revealed by the analysis of engineered *Clcn7*^−/−^ mice (Kornak et al., 2001) and the *grey-lethal* mouse line, which was found to harbor a mutation leading to Ostm1 deficiency (Chalhoub et al., 2003). Homozygous mice of both lines develop a severe osteopetrosis, i.e., hypermineralization of bones and obliteration of bone marrow cavities, accompanied by secondary effects such as a lack of tooth eruption. The osteopetrosis is owed to an impairment in the build-up and in the acidification of the osteoclast ruffled border (Kornak et al., 2001; Rajapurohitam et al., 2001; Stauber et al., 2012; Jentsch and Pusch, 2018). The mice display a shortened life span of only few weeks. In an *agouti* background, when wild-type mice have brown fur, ClC-7- and Ostm1-deficient mice have a gray coat color, suggesting a role for ClC-7/Ostm1 in hair pigmentation (Kornak et al., 2001; Chalhoub et al., 2003).

Besides the immediately obvious osteopetrotic phenotype, *Clcn7*^−/−^ and *grey-lethal* mice develop a progressive neurodegeneration in the brain and retina (Kasper et al., 2005; Lange et al., 2006; Pressey et al., 2010). Neurons of various brain regions accumulated electron-dense deposits in lysosomes scattered throughout the cell bodies (Kasper et al., 2005). With the autofluorescence of the lysosomal storage material and the accumulation of the subunit c of the mitochondrial ATP synthase, the phenotype resembled a neuronal ceroid lipofuscinosis (NCL). Neuronal cell loss, prominent in the hippocampal CA3 region, in the thalamocortical system and of Purkinje cells in the cerebellum, was accompanied by inflammatory responses such as microglia activation and astrogliosis, another hallmark of NCL and other neurodegenerative pathologies, in *Clcn7*^−/−^ mice (Kasper et al., 2005; Pressey et al., 2010). The generation of tissue-specific ClC-7 knock-out mice enabled the analysis of neurodegeneration in mice with a normal life span (Wartosch et al., 2009). Brains of adult mice with a forebrain-specific ClC-7 knock-out (*Clcn7*^lox/lox^*;EMX1-cre*), displayed conspicuous loss of hippocampal and cortical neurons. Starting in the CA3 region of hippocampus neuronal loss progressed into the dentate gyrus and by the age of 1.5 years no hippocampal structures in *Clcn7*^lox/lox^*;EMX1cre* mice could be detected (Wartosch et al., 2009). *In vivo* protein degradation experiments using kidney-specific ClC-7 knock-out mice (*Clcn7*^lox/lox^*;ApoE-cre*) revealed slowed lysosomal degradation of endocytosed protein (Wartosch et al., 2009). Consistent with an impairment of lysosomal function, *grey-lethal* mice accumulated sphingolipids in the brain (Prinetti et al., 2009) and an increase in the autophagic marker LC3-II was observed in brain and kidney of *Clcn7*^−/−^ and in *grey-lethal* mice (Wartosch et al., 2009; Heraud et al., 2014), but it is not clear whether this is due to reduced autophagosome clearance or an induction of autophagy (Wartosch and Stauber, 2010).

Lysosomes in neurons and other cells from ClC-7- or Ostm1-deficient mice were found to be normally acidified (Kasper et al., 2005; Lange et al., 2006; Steinberg et al., 2010), contradicting the previous assumption that ClC-7 mediated the required counterion transport for the lysosomal acidification. Instead, the counterion conductance can be provided by cation efflux from lysosomes (Steinberg et al., 2010; Weinert et al., 2010). *Clcn7*^unc/unc^ mice, in which ClC-7 functions as a pure Cl^−^ conductor due to the E245A mutation of its “gating glutamate” did not display a fur color phenotype and they presented an osteopetrosis that was milder than in *Clcn7*^−/−^ mice (Weinert et al., 2010). However, they developed the same lysosomal storage disease and neurodegeneration as ClC-7 knock-out mice (Table 2). As in in *Clcn7*^−/−^ cells, lysosomes were acidified to the normally low pH. As predictable from model calculations (Weinert et al., 2010; Ishida et al., 2013; Astaburuaga et al., 2019), the lysosomal Cl^−^ concentration was reduced in both *Clcn7*^−/−^ and *Clcn7*^unc/unc^ mice compared to wild-type (Weinert et al., 2010). Similar results, reduced [Cl^−^] but normally acidic pH, were obtained with nematode models with reduced ClC-7 or Ostm1 orthologs (Chakraborty et al., 2017). Together with the equivalent ClC-3^unc^ and ClC-5^unc^ knock-in mouse models, this suggests an important, acidification-independent role for Cl^−^ accumulation in the endo-lysosomal pathway, while in early endosomes Cl^−^ additionally supports luminal acidification (Novarino et al., 2010; Weinert et al., 2010, 2020; Scott and Gruenberg, 2011; Stauber and Jentsch, 2013; Schwappach, 2020).

Another knock-in mouse model expressed ClC-7 with a E312A mutation of the “proton glutamate,” dubbed ClC-7^td^ because of the supposed *t*ransport *d*eficiency of the mutant (Weinert et al., 2014) (Table 2). Like *Clcn7*^unc/unc^ mice, these *Clcn7*^td/td^ mice lacked a coat color phenotype. However, in contrast to *Clcn7*^unc/unc^ mice, the osteopetrosis of *Clcn7*^td/td^ mice was as pronounced as in *Clcn7*^−/−^ mice, but intriguingly their neuropathy with lysosomal storage and neurodegeneration was less severe and no accumulation of autophagic material was detected (Weinert et al., 2014). An explanation for the differential effects in the mouse models may be that the Cl^−^ conductance of ClC-7^unc^ could partially compensate for ClC-7 loss in its osteoclast function in bone resorption, while it is deleterious for neuronal lysosomes. On the other hand, the presence of the ClC-7^td^ protein–and with that of Ostm1 which is normally transported to lysosomes in *Clcn7*^td/td^ mice (Weinert et al., 2014)–can sustain lysosomal function in neurons, possibly by protein-protein interactions as proposed for Ostm1 (Pandruvada et al., 2016). An alternative explanation for the milder neuropathy of *Clcn7*^td/td^ mice compared to *Clcn7*^−/−^ mice was recently provided by the unexpected finding that the mutation of the “proton glutamate” to alanine does not completely abolish currents by ClC-7^td^ (Pusch and Zifarelli, 2021). With its residual ion transport activity, ClC-7^td^ may partially exert its function in the neuronal endo-lysosomal pathway. Nonetheless, while lysosomes of *Clcn7*^td/td^ mice were normally acidified, the lysosomal Cl^−^ concentration was reduced (Weinert et al., 2014).

### Patient *CLCN7* Mutations Causing Neuropathies

Mutations in *CLCN7* and *OSTM1* also underlie severe, autosomal recessive osteopetrosis (ARO) in humans (Kornak et al., 2001; Chalhoub et al., 2003). In addition, intermediate autosomal osteopetrosis (IAO) and the milder, autosomal dominant osteopetrosis type 2 (ADO2, or Albers-Schönberg disease) are caused by *CLCN7* mutations (Cleiren et al., 2001; Frattini et al., 2003; Sobacchi et al., 2013). The most severe forms of *CLCN7*-related ARO in about 50% of the cases, as well as *OSTM1*-related ARO, are also neuropathic with primary neurodegeneration manifesting in developmental delay, hypotonia, retinal atrophy and seizures (Steward, 2003; Pangrazio et al., 2006; Sobacchi et al., 2013), which is critical for therapy (Teti and Econs, 2017). Dominant *CLCN7* mutations, which may lead to subcellular mislocalization of the ClC-7/Ostm1 complex or the impingement of the dysfunctional subunit on the ion transport properties of the unaffected subunit (Schulz et al., 2010; Ludwig et al., 2013), do not lead to neuropathy in mild ADO2. A mouse model recapitulating the most common ClC-7 mutation in ADO2, G215R (G213R in mouse), which was reported to impinge on subcellular trafficking (Schulz et al., 2010), developed a late-onset osteopetrosis as expected for heterozygous animals while homozygous mice displayed a phenotype similar to that of *Clcn7*^−/−^ mice with death after only a few weeks and neurodegeneration (Alam et al., 2014) (Table 2). Similar findings were made with another ADO2 mouse model with the F318L (F316L in mouse) mutation (Caetano-Lopes et al., 2017), which strongly diminishes currents mediated by ClC-7^PM^/Ostm1 (Leisle et al., 2011). However, heterozygous *Clcn7*^+/G213R^ ADO2 mice also presented fibrosis in non-skeletal tissues such as lung and muscle; their brains exhibited perivascular fibrosis, β-amyloid accumulation and astrogliosis, and the animals showed behavioral abnormalities (Maurizi et al., 2019).

During the first electrophysiological analysis of osteopetrosis-causing ClC-7 missense mutations, it was surprisingly found that, besides loss-of-function mutations due to impaired ER exit or reduced ion transport, several pathogenic ClC-7 mutations accelerated the voltage-dependent activation of ClC-7^PM^/Ostm1 (Leisle et al., 2011). Most of the amino acids changed by these mutations are involved in the interaction between the transmembrane domain and the cytoplasmic CBS domains of ClC-7, which highlights the role of the domain interface in the common gating that is responsible for the slow activation kinetics (Leisle et al., 2011; Ludwig et al., 2013; Schrecker et al., 2020; Zhang et al., 2020). Such apparent gain-of-function by fast exchanger activation was subsequently shown for further mutants (Barvencik et al., 2014; Sartelet et al., 2014; Di Zanni et al., 2020). There seems to be no strict correlation between the effect a given mutation exerts on the current properties–whether it diminishes currents or accelerates the voltage-dependent activation–and the neuropathy of osteopetrosis. Instead, it was recently proposed that the mere presence of ClC-7/Ostm1 may determine the severity of the disease (Di Zanni et al., 2020), but the genotype-phenotype correlation is far from clarified.

Recently, a heterozygous *de novo* mutation of ClC-7 was identified in two children with hypopigmentation and delayed development (Nicoli et al., 2019). The patients exhibited organomegaly and their brains displayed delayed myelination, a thin posterior corpus callosum, hyperintensity of the subthalamic nuclei, and cerebellar atrophy in MRI. Remarkably, they had no signs of osteopetrosis. Huge intracellular vacuoles and storage material was found in various tested tissues. The phenotype of knock-in mice heterozygous for the identified Y715C (Y713C in mouse) mutation recapitulated the disease (Nicoli et al., 2019) (Table 2). Electrophysiological characterization of the mutant revealed a tremendous increase in current amplitude. Heterologous expression of ClC-7^Y715C^ led to a drastic enlargement of late endosomal/lysosomal compartments (Nicoli et al., 2019), similar to those with the gain-of-function ClC-6^Y553C^ mutant (Polovitskaya et al., 2020). In the case of ClC-7^Y715C^, the enlarged compartments were reported not to be acidified in contrast to surrounding smaller, hyperacidified lysosomes, which was attributed to the hyperactivity of the ClC-7 mutant (Nicoli et al., 2019). The mechanism of the endosomal/lysosomal enlargement remains elusive. This mutant corroborates the notion that osteopetrosis and neuropathy with ClC-7 mutations may develop with independent pathomechanisms as ClC-7 may exert cell type-specific functions.

## Conclusions

Loss-of-function and in some cases also gain-of-function of the intracellular CLCs, ClC-3/ClC-4, ClC-6 and ClC-7/Ostm1, lead to neuropathies, often neurodegeneration. They all function as Cl^−^/H^+^ exchangers on distinct, but overlapping organelles of the endosomal-lysosomal pathway (Stauber et al., 2012; Jentsch and Pusch, 2018). Trafficking and function of these compartments is detrimental for cellular physiology and dysfunction of the degradative pathway, lysosomal clearance by exocytosis or autophagic delivery to this pathway are associated with neurodegenerative disorders (Hara et al., 2006; Komatsu et al., 2006; LaPlante et al., 2006; Nixon, 2013; Wang et al., 2013; Menzies et al., 2017; Ballabio and Bonifacino, 2020; Mallucci et al., 2020).

In some cases, it has been proposed that the CLC serves as a structural protein that functions to recruit other proteins such as components of the transport machinery by direct protein-protein interaction. Amongst others, the existence of different missense mutations which unlikely all prevent the same interactions argues against this. Although a role a role for protein-protein interactions of intracellular CLCs in vesicular trafficking cannot be excluded, the most straight-forward explanation for the importance of CLCs is their role in the regulation of the vesicular ion homeostasis. In this respect, the pronounced outward rectification of the vesicular CLCs remains enigmatic, because it would strongly favor Cl^−^ export from endosomes/lysosomes. At membrane voltages in the range of those measured for endosomal/lysosomal compartments, which can be up to inside-positive 100 mV (Koivusalo et al., 2011; Saminathan et al., 2021), the CLC exchangers are virtually inactive in heterologous expression systems (Friedrich et al., 1999; Li et al., 2000; Neagoe et al., 2010; Leisle et al., 2011). Nonetheless, the effects of CLC depletion or various mutations -both loss and gain of function- demonstrate Cl^−^ accumulation by CLCs and the necessity of their ion transport. Mutation of the “gating glutamate” does not only convert the exchanger into a pure Cl^−^ conductor, but also abolishes its voltage dependence. Therefore, such a mutation may indeed represent a gain of function in respect to Cl^−^ transport. Interestingly, while the uncoupling mutation of ClC-5 or ClC-7 leads to effects that are similar to the respective knock-out in mouse models, the heterozygous mutant of ClC-6 results in a much more severe disorder in patients than in the murine gene knock-out model (Poët et al., 2006; He et al., 2021).

The luminal pH is of undisputed importance for endosomes and lysosomes and impaired acidification is linked to neurodegeneration (Mellman et al., 1986; Mindell, 2012; Song et al., 2020). However, while the early endosomal ClC-5 critically supports acidification by providing the counterion (Günther et al., 1998; Piwon et al., 2000), this does not seem to be the case for ClC-3/-4, ClC-6 or ClC-7 on later endosomes and lysosomes (Kasper et al., 2005; Poët et al., 2006; Weinert et al., 2020) where the larger cation conductance can support acidification (Van Dyke, 1993; Steinberg et al., 2010). Instead, analyses of various mouse models have shown that the secondary active, pH-gradient driven accumulation of Cl^−^ by their Cl^−^/H^+^ exchange mechanism is pivotal –also for ClC-5, in addition to the acidification- (Novarino et al., 2010; Weinert et al., 2010, 2020). Along the endosomal-lysosomal pathway, the luminal Cl^−^ concentration rises to > 100 mM in the lysosome (Saha et al., 2015). Reduced lysosomal Cl^−^ concentrations, but a normally low pH, have been found in nematode models of various neurodegenerative lysosomal storage diseases including Gaucher and Nieman-Pick A and B (Chakraborty et al., 2017). The Cl^−^ concentration has been shown to influence the enzymatic activity of the lysosomal protease cathepsin C (Cigić and Pain, 1999), but the general mechanism by which luminal Cl^−^ affects vesicular function is elusive (Stauber and Jentsch, 2013).

**Figure 3:**
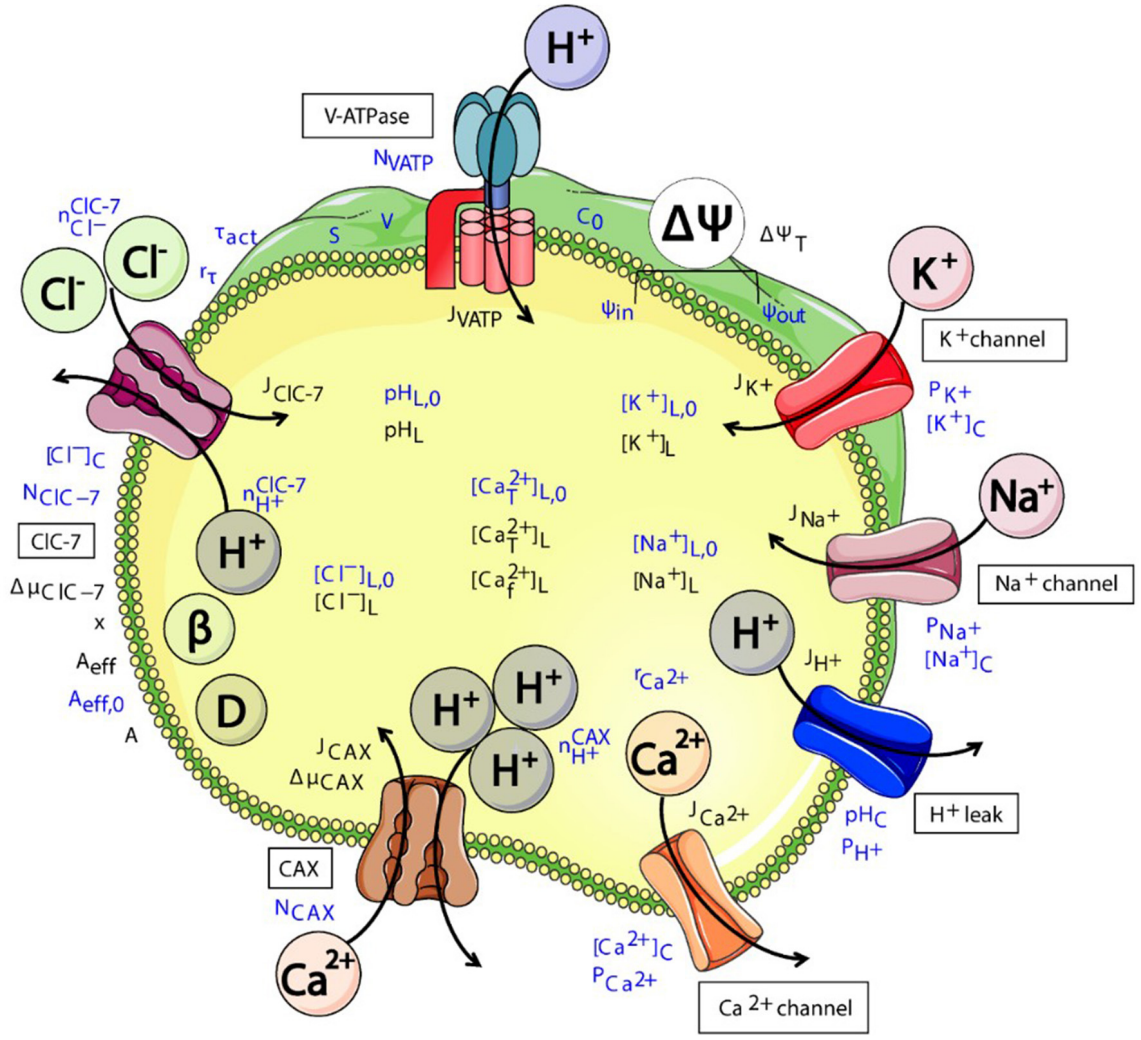
Schematic representation of various (partially putative) components of the interconnected system of ensosomal/lysosomal ion homeostasis. CLC exchanger activity (here exemplary of ClC-7) directly affects the luminal pH and Cl^−^ concentration. These will affect the transmembrane voltage ΔΨ and concentrations of further ion species and hence osmolarity. The cartoon was created using Servier Medical Art templates (https://smart.servier.com) licensed under a Creative Commons License (https://creativecommons.org/licenses/by/3.0/). The figure was taken from Astaburuaga et al. (2019).

The presence of a Cl^−^/H^+^ exchanger does not only –if at all– affect the luminal Cl^−^ concentration and pH, but ion homeostasis in general, including the transmembrane voltage and concentrations of other ion species (Ishida et al., 2013; Stauber and Jentsch, 2013; Astaburuaga et al., 2019) (Figure 3). One particularly important ion species that may be affected is Ca^2+^ (Chakraborty et al., 2017; Astaburuaga et al., 2019), as it is crucially involved in signaling and trafficking in the endosomal-lysosomal pathway (Luzio et al., 2007; Morgan et al., 2011; Ureshino et al., 2019; Lakpa et al., 2020). In addition, the exchange activity of CLCs has osmotic effects on the respective organelles. The enlargement of late endosomal/lysosomal compartments upon overexpression of ClC-3 (Li et al., 2002) or with gain-of-function mutants of ClC-6 or ClC-7 (Nicoli et al., 2019; Polovitskaya et al., 2020), may be partially due to osmotic swelling. Furthermore, osmotic volume changes promote membrane trafficking events (Freeman and Grinstein, 2018; Freeman et al., 2020; Saric and Freeman, 2020) and the number of known osmo-sensitive cation channels of endosomes and lysosomes is growing (Chen et al., 2020a,b). Recently, the LRRC8-formed volume-regulated anion channel (VRAC) has been reported to localize to lysosomes where it contributed to cellular osmoreglation (Li et al., 2020). This plasma membrane channel, which is involved in multiple physiological processes, does not only mediate cell volume regulatory ion transport (Chen et al., 2019). By releasing glutamate from swollen cells under ischemic conditions, it contributes to excitotoxicity and neuronal cell death after stroke (Mongin, 2016; Yang et al., 2019).

In summary, dysfunction of the intracellular CLC Cl^−^/H^+^ exchangers impinges on the trafficking, function and possibly signaling of endosomal and lysosomal organelles and interconnected pathways such as autophagy, which in turn leads to cell death of vulnerable neurons. Future work is required to uncover in detail the molecular mechanism by which these CLCs contribute to the functioning of the endosomal-lysosomal pathway.

## Author Contributions

SB, HH, and TS wrote the manuscript. All authors contributed to the article and approved the submitted version.

## Conflict of Interest

The authors declare that the research was conducted in the absence of any commercial or financial relationships that could be construed as a potential conflict of interest.
